# Experiências d’“Áfricas”: coleções de peças africanas de três artistas portugueses contemporâneos

**DOI:** 10.1590/S0104-59702025000100034

**Published:** 2025-08-25

**Authors:** Jorge Croce Rivera

**Affiliations:** i Investigador, IN2PAST/Centro de História de Arte e Investigação Artística/Universidade de Évora. Évora – Portugal rivera@uevora.pt

**Keywords:** Cruzeiro Seixas (1920-2020, Eduardo Nery (1938-2013, José de Guimarães (1939, África, Coleções de arte, Cruzeiro Seixas (1920-2020, Eduardo Nery (1938-2013, José de Guimarães (1939, Africa, Art collections

## Abstract

Antiga potência colonial, são frequentes em Portugal coleções privadas de peças oriundas das suas possessões em África. Reunidas, em geral, por membros da administração colonial, militares, comerciantes, simples colonos ou viajantes, hoje guardadas pelos seus herdeiros, elas encontram-se também em outros grupos: intelectuais, especialistas, empresários, galeristas ou artistas, entre outros. Neste estudo são consideradas as coleções de “arte africana” de três artistas contemporâneos de diferentes orientações estéticas – Cruzeiro Seixas, Eduardo Nery e José de Guimarães –, atentando-se às suas vivências e representações de “África”, aos modos de constituição das coleções e ao influxo das peças no percurso pessoal e na obra de cada um desses artistas.

## Abstract (en)

Because of its colonial history, Portugal holds many collections of African objects from its former possessions. They were generally gathered by colonial administrators, the military, merchants, simple settlers or travelers, and today are maintained by their heirs, but are also found among other groups including intellectuals, specialists, entrepreneurs, gallery owners and artists. This study looks at collections of “African art” belonging to three contemporary artists with different aesthetic orientations (Cruzeiro Seixas, Eduardo Nery and José de Guimarães) and considers their experiences and representations of “Africa,” how the collections came to be created, and the influence of these pieces on the personal career and work of each of these artists.

Apesar das coleções de peças extraeuropeias, existentes em instituições portuguesas desde a segunda metade do século XVIII, terem sido estudadas em anos recentes – sejam as coleções de âmbito “palaciano”, no Real Gabinete da Ajuda, “universitário”, no Museu de História Natural de Coimbra, “académico”, no Museu da Real Academia de Ciências de Lisboa, ou mais genericamente “científico”, na coleção da Sociedade de Geografia de Lisboa, no Museu Nacional de Arqueologia ou no Museu Santos Rocha, na Figueira da Foz (Cantinho, 2010) –, permanecem relativamente desconhecidas as coleções de peças extraeuropeias na posse de pessoas singulares.

Não é excecional em Portugal a presença, em residências particulares, de peças oriundas das costas de África, Brasil, Índia, Macau e Timor: estatuetas e relicários, marfins talhados, mobiliário, utensílios, tecidos, cestaria, armas, peças decorativas, trazidas para a Europa desde o século XVI por marinheiros e soldados, figuras da administração portuguesa, religiosos, técnicos, comerciantes, colonos ou simples viajantes. Em algumas das coleções institucionais acima referidas, encontram-se peças que foram ofertas de militares ou administradores, no regresso de estadias nas colónias.

Se essas peças são, em geral, valiosas, e os particulares que as possuem de estatuto social elevado, a intensificação da ocupação colonial no século XX, a deslocação de contingentes de militares para as “províncias ultramarinas” durante as guerras coloniais em África, entre 1961 a 1974, e o regresso dos colonos no início do processo de descolonização explicam igualmente a existência de peças africanas – estatuetas, máscaras, instrumentos musicais, armas tradicionais – em residências de grupos e estratos sociais de menor relevância social. Em muitas casas de classe média, peças extraeuropeias surgem dispostas como decorativas, combinadas com peças de arte e mobiliário ocidentais, por vezes, a par de peças adquiridas em viagens turísticas recentes.

Todavia, para além dessas peças, soltas e, em geral, de qualidade mediana, que não constituem propriamente coleções, mas *memorabilia* ou decoração, encontram-se conjuntos, por vezes amplos, outros seletivos, de peças de grande qualidade. Alguns desses acervos são antigos, outros de constituição mais próxima, pertencentes, em alguns casos, a herdeiros de antigos administradores, aristocratas ou militares, noutros, a particulares que se não reclamam desse passado, mas são intelectuais, artistas, galeristas, profissionais liberais, técnicos de instituições internacionais, financeiros ou empresários.

Em anos recentes, particulares estrangeiros, entre eles comerciantes africanos, instalaram-se no país e abriram galerias especializadas, negociando peças trazidas de vários pontos de África, sobretudo da costa ocidental. Generalizou-se também a compra e venda por meio de leilões *on-line*. A situação é fluída, peças de qualidade assinalável ou mediana surgem em feiras e mercados de arte, vendidas em galerias e antiquários, disputadas em casas de leilões, num processo dinâmico de circulação internacional de peças que ultrapassa os grupos sociais e o mercado português, nele refletindo-se a cosmopolitização de Portugal após a sua integração na União Europeia, em 1986.

Se a constituição das coleções institucionais tem genericamente uma justificação científica, como determinar as razões do colecionismo por particulares? Por que motivações íntimas ou razões estéticas, pelo prestígio da posse ou por emulação social, por investimento financeiro ou especulativo? Quais os atores e os modos de criação, de circulação, de aquisição? Quais os critérios de coleção, como são preservadas e expostas as peças? Qual a sustentabilidade económica dessas coleções? Apoiam-se os colecionadores em alguma instituição privada ou no Estado? Tem o Estado interesse na preservação dessas coleções? Qual a relação que os colecionadores estabelecem, social e profissionalmente, com essas coleções?

O horizonte de pesquisa que essas coleções solicitam é deveras amplo e complexo, há múltiplos aspetos, registos e variáveis cujos reconhecimento e operacionalidade nos escapam inteira ou parcialmente. Sob a aparente evidência de serem um reflexo do estatuto social e do poder económico dos colecionadores, as coleções reflectem as idiossincrasias e as motivações dos colecionadores, ocorrem em contextos inteiramente diversos, jogando-se neles circunstâncias e oportunidades, a compulsão ao colecionismo e a emulação entre colecionadores.

Contudo, se essas questões se poderão igualmente colocar em outros contextos, pelo menos europeus, em que medida a concreta história social e política portuguesa se implica com essas coleções? Sendo de peças extraeuropeias, de que modo se descobrem aspetos concretos do passado colonial português; em particular, do colonialismo do século XX, em que ocorreram insurreições armadas que levaram à independência das colónias e ao regresso forçado dos colonos? De que modo reflectem a situação pós-colonial, seja o confinamento de Portugal ao espaço europeu imediatamente após 1974, depois, a sua integração na União Europeia e, finalmente, a inserção de Portugal numa dimensão global e cosmopolita?

No processo de estudo dos colecionadores privados, impôs-se como singular a coincidência de três artistas portugueses – Cruzeiro Seixas (1920-2020), Eduardo Nery (1938-2013) e José de Guimarães (1939- ) –, dos mais relevantes na arte contemporânea portuguesa, terem constituído importantes coleções de peças africanas no período de transformações políticas e sociais que marcaram Portugal desde a Segunda Guerra Mundial. Cruzeiro Seixas, jovem surrealista, vendedor ambulante no interior de Angola nos anos 1950, por meio de recolha direta junto das populações; Eduardo Nery, expoente do Modernismo geométrico abstrato, adquirindo peças a comerciantes e galeristas, desde a Exposição Universal de Lisboa, em 1998, até ao seu falecimento, em 2013; José de Guimarães, promotor de rejuvenescimento da *pop art* como arte global, primeiro em Angola, como oficial do Exército, entre 1967 e 1974, depois na Europa, dos anos 1980 até a atualidade.

Constituídas em circunstâncias muito diversas por esses artistas, não como etnógrafos, mas enquanto as peças lhes despertavam a inteligência, a imaginação e a sensibilidade, as coleções apontam para experiências intensas e diversas da realidade antropológica africana. Como um suposto que perpassa todas as peças reunidas, “África” surge-lhes revestida de irredutível alteridade, um “amor correspondido” que deslumbra e desconcerta, com quem se estabelece uma relação fascinada, por vezes obsessiva, sempre dramática. “África” seria o “último continente surrealista” (Seixas), a possibilidade de um encontro com “a energia e as formas de vida” (Nery), em cuja arte opera um “alfabeto” que abre caminho à descoberta de “alfabetos” noutras culturas (Guimarães).

As experiências de “África” revelam-se momentos decisivos nos seus percursos pessoais, mas foram-no de modo idêntico? De que modo a intensidade dessas experiências foi adaptada ao quadro “objetivo” da estética ocidental, como a realidade antropológica de “África” afetou a produção e as posições artísticas de cada um destes artistas? E, de que modo se joga neles a história mesma das correntes estéticas que integraram? Como lhes terá surgido o interesse pelas peças africanas? Repetiam conscientemente a atitude de Picasso, Derchain e Max Ernst, prosseguiam a atenção de Bréton ao primitivismo, ou as razões fundas que assistiram às suas coleções eram-lhes idiossincráticas?

De que modo Cruzeiro Seixas assumiu ali a racionalidade “extracartesiana” do surrealismo? De que formas a desconstrução da perceção visual, o rigor geométrico e a fantasia e humor da estética de Eduardo Nery se puderam conciliar com a vitalidade das peças da sua coleção africana? O engenho dos provérbios esculpidos nas tampas de Ngoyo, nos quais José de Guimarães procurava encontrar os signos primordiais, é reconhecível na platitude combinatória dos seus “alfabetos”? Na realidade, que “Áfricas” estão supostas nessas coleções?

E, tendo vivido uma parte significativa imersos em situações políticas, não apenas autoritárias, mas coloniais, de que modo tal condição histórica se refletiu nas coleções? Anteviram ou entreveram a questão da “colonialidade”, de uma pretensão ou presunção de superioridade civilizacional ocidental, ou a existência de uma relação assimétrica com as populações africanas – e por extensão também dos objetos? Que tipo de conhecimento lhes assistiu nas suas coleções, que análises puderam realizar das peças? De que modo as guardaram e preservaram? Quais os destinos das suas coleções?

Assim, de um modo por vezes inesperado, confluem nessas três coleções a diversidade e complexidade de problemas que tínhamos entrevisto na consideração das outras coleções privadas, descobrindo no seu estudo conjunto a possibilidade de construção de uma matriz de aspetos, registos e variáveis que julgamos conveniente para o estudo das demais alargadas coleções.

Mais que enquadrar os três casos num modelo interpretativo, o artigo tentou detetar os aspetos e as variáveis que importam à reconstituição do significado e do valor dessas coleções, de modo que essa matriz de equações possa ser alargada ao estudo de outras coleções, sem se perder a densidade e a complexidade singular dos atores e dos processos, dos acervos e das peças.

Evitando uma perspetiva judicativa ou a adoção de um modelo teorético, o estudo assumiu a exigência fenomenológica da suspensão de juízo, privilegiando a descrição minuciosa dos contextos, características e destino de cada coleção. Com o objetivo de dotar o estudo de coerência problemática, a exposição das linhas de pesquisa foi feita em função da referência a “África”, não a tomando como uma entidade geográfica e antropológica concreta, nem como motivo literário (Ribeiro, 2017), mas como uma entidade enigmática, densa e complexa, que surge a cada um dos artistas conjugando dimensões reais, simbólicas e imaginárias. No título do artigo, o uso do apóstrofe e do plural indicia múltiplas relações, cada uma delas indiciando a relação de cada artista com um *ethos* envolvente, que entrevê apenas parcelarmente. E, reversivamente, os diferentes registos que compõem a realidade antropológica “africana” relacionam-se multimodamente com cada artista, nunca se deixando inteiramente objetivar.

O estudo resulta de várias linhas de pesquisa, antes de mais, nos três núcleos do espólio de Cruzeiro Seixas, na Biblioteca Nacional de Portugal, em Lisboa, na Fundação Cupertino de Miranda, em Famalicão, e no Arquivo Histórico da Universidade de Évora. As reflexões autobiográficas dos artistas, expostas em catálogos ou entrevistas, foram as referências fundamentais, que foram complementadas pelas perspetivas dos críticos e conviventes dos artistas.

Contactámos o artista José de Guimarães, os familiares de Eduardo Nery e amigos próximos de Cruzeiro Seixas, os familiares do empresário Manuel Vinhas, cuja importância assinalaremos, além de vários dos antiquários em Lisboa e no Porto, comerciantes africanos, alguns colecionadores e artistas que conjugam todas essas vertentes – colecionam, comercializam e permutam.

Não foi possível o acesso à coleção de Eduardo Nery, atualmente integrada na Associação Coleção Berardo, mas o responsável, Álvaro Silva, respondeu amavelmente a todas as questões que lhe foram colocadas. Pudemos ver, na Fundação Cupertino de Miranda, em Famalicão, em visita em novembro de 2023, por amabilidade da sua responsável, Marlene Silva, o conjunto das peças etnográficas que faziam parte do espólio ali depositado. Encontrámos igualmente respostas dos responsáveis institucionais a todas as questões sobre a doação que José de Guimarães fez ao Museu Martins Sarmento, em Guimarães, em 1970, e tomámos conhecimento, por meio da diretora Marta Mestre, das estratégias curatoriais para os depósitos de José de Guimarães no Centro Internacional de Arte José de Guimarães, realizados em 2010 e 2020. A todos, expressamos o nosso agradecimento.

## Cruzeiro Seixas

A primeira das coleções a considerar é a de Artur de Cruzeiro Seixas, resultante de uma recolha em vários pontos de Angola e Cabinda, entre 1953 e 1960, durante os últimos anos do período colonial português. Realizada durante viagens do artista como vendedor ambulante, a recolha não teve uma intenção científica, mas nasceu de uma forte experiência humana com as populações e da admiração pelas qualidades das peças. Essa coleção não pode ser separada da personalidade e do percurso artístico de Cruzeiro Seixas, poeta e artista plástico que desempenhou um papel fundamental no movimento surrealista em Portugal.

Falecido em 2020, tendo vivido quase até aos cem anos, evocava a sua estada em África, entre 1952 e 1964, como o momento decisivo no seu percurso pessoal e artístico, correspondente a um lugar cosmopolita: “África foi o meu Paris”, dizia.

Oriundo de uma família tradicional do Norte de Portugal, republicana e abastada, mas que tinha sofrido um forte revés económico, filho muito estimado de pais tardios, homossexual assumido, mas discreto, Cruzeiro Seixas estudou em Lisboa, na Escola António Arroio, um estabelecimento dedicado ao ensino artístico. Em meados da década de 1940 experimentou vários estilos, aproximou-se do neorrealismo, do qual se afastou ao adotar os princípios do surrealismo. Começou a participar ativamente nas atividades dos surrealistas no final da década de 1940; membro do grupo liderado por Mário Cesariny, do qual faziam parte artistas como António Maria Lisboa, Mário-Henrique Leiria, Pedro Oom e Carlos Calvet, participou nas exposições do grupo em 1949 e 1950 e colaborou em vários manifestos e intervenções políticas, numa atitude de forte crítica a quaisquer formas de autoritarismo e moralismo, fossem de direita ou de esquerda.

Em 1950, alistou-se na Marinha Mercante e percorreu, durante dois anos, os portos das antigas colónias portuguesas em África, Ásia e Timor. Em 1952, instalou-se em Luanda, primeiro no palácio do governador-geral, seu parente, e, um ano depois, num apartamento com os pais, que se lhe tinham juntado. Desgostando-lhe a vida urbana colonial, percorreu o interior de Angola e de Cabinda em funções pouco qualificadas – vendedor ambulante de rádio-transistores ou apólices, pintor de painéis publicitários. As condições que encontrava nessas longas viagens eram muito precárias, percorrendo estradas deficientes, sujeitas a intempéries, as suas atividades suspeitas aos olhos das autoridades, vivendo situações imprevisíveis que o obrigavam, não apenas a adaptar-se a condições difíceis, mas a contactar intensamente com as populações. Atento aos modos múltiplos do colonialismo, nunca se esquecendo da sua situação de europeu, Cruzeiro Seixas conseguiu um conhecimento ímpar do território e das populações (Seixas, 1989, p.136; Pereira, 2011, p.704).

Nessas viagens, ele foi assistindo a cerimónias rituais, registando costumes e indumentárias, tipos de construção e cenas de trabalho, pinturas nas paredes e sessões musicais. Cruzeiro Seixas fotografava também a arquitetura civil e militar colonial – fortes e casas de administração – e recolhia documentação histórica sobre o colonialismo e as práticas escravizantes: postais, listas de trabalhadores, de distribuição de água, pedidos para a realização de festas familiares etc. Convivendo com autoridades locais, portuguesas e angolanas, captava também cenas de conflitos abertos entre os fazendeiros portugueses e as comunidades indígenas, como as fotografias de uma “sanzala”, aldeia incendiada pelos fazendeiros, em Quicolongo, no Norte de Angola, em agosto de 1958 (BNP ESP N38/1677-1684, s.d.).

Experimentando ao mesmo tempo a solidão, a vida aventurosa e a sublimidade dos lugares – “Em África a paisagem olha-nos” –, começa a escrever poesia, assinando os textos como tendo sido escritos em “Áfricas”, no plural, de modo a diferenciar a “África” como território geográfico, dominado mas escassamente conhecido dos europeus, e a “África” como a realidade em que ali viviam os humanos.

Durante as viagens ao interior, pintava aguarelas de cores intensas e figuras abstratas, que denomina genericamente “Paisagens de África”, ao mesmo tempo que continuava a criar pinturas e desenhos de temática imaginária, com um traço muito fino e preciso, figuras oníricas colocadas como que esculturas numa paisagem metafísica, também colagens e peças escultóricas surrealistas, voltadas para a crítica ao colonialismo, ao moralismo e às convenções sociais (Letria, 2020, p.73-74).

Mantendo sempre contacto com os amigos surrealistas em Portugal e ilustrando obras que iam sendo publicadas em Lisboa, organiza em Luanda duas exposições surrealistas de pintura, desenhos e objetos, ambas de temática explicitamente anticolonial. A primeira, realizada em 1953 num *foyer* de um cinema em Luanda a que a população africana não tinha acesso, mostrava desenhos e aguarelas, sob a evocação de um verso de Aimé Cesaire – “Je retrouverais le secret des grandes communications et des grandes combustions. Je dirais orage. Je dirais fleuve. Je dirais tornade. Je dirais feuille. Je dirais arbre” [Irei redescobrir o segredo das grandes comunicações e das grandes combustões. Eu direi tempestade. Eu direi rio. Eu direi tornado. Eu direi folha. Eu direi árvore] (BNP N38/1158-1159, 1953) –, referência que o levou a ser interrogado pela polícia política. A exposição, provavelmente a primeira de intento surrealista realizada em África, provocou intensa polémica nos jornais, dividindo críticas. De Lisboa, recebeu o apoio de Mário Cesariny, que, numa entrevista, afirmou:

A África é o último continente surrealista. Tudo o que antecede, combate ou ultrapassa a interpretação estreitamente racionalista do homem e dos seus modos tem a ver com um sentido surrealista da vida. A África goza do privilégio raro de não ter produzido nem cartesianismo nem os sistemas de pensamento e ação baseados em universos de categorias. A África conhece um mito que nós ignoramos. Julgamo-la adormecida no passado e está talvez perfazendo o futuro (BNP ESP N38/1108-1108A, 1954).

Após essa exposição, Cruzeiro Seixas começa a recolher peças etnográficas durante as viagens no interior de Angola: peças de uso quotidiano ou de prática ritual, estatuetas, máscaras, armas, vasilhas, instrumentos musicais, esteiras, pentes, portas esculpidas e pintadas, entre outras, recolhidas por toda Angola, de Cabinda ao Cunene. Provavelmente terá sido essa também a ocasião de leituras de antropologia, certamente de Lévi-Strauss e que privou com investigadores do Museu de História Natural de Angola; em particular, com Carlos Coimbra, notável erudito, conservador e investigador no Museu.

Em 1957, realizou uma segunda exposição, para a qual teve a colaboração de dois jovens intelectuais, o casal Alfredo e Manuela Margarido, ambos funcionários públicos. Concebida sob o signo de Lautréamont como uma instalação nas ruínas de um palácio do século XVIII, no centro de Luanda, chamado o “Palácio dos Fantasmas”, a mostra apresentava pinturas, colagens, desenhos, aguarelas, esculturas, e também estatuetas de arte africana, tudo envolto num cenário de redes e correntes que deveria sugerir aos visitantes a sensação de estarem debaixo das águas do oceano, simbolizando a imersão na situação colonial, abaixo da verdadeira vida à superfície (BNP ESP N38/1160, 1964). Tal como a anterior, a exposição, inaugurada pelo secretário-geral e elogiada pelo governador-geral, como sinal de modernidade cultural, e muito visitada pelas populações europeia e africana, teve uma enorme repercussão, em geral negativa, nos jornais de Angola, com artigos críticos e inquéritos, nos quais intervieram também intelectuais em Portugal. Das posições assumidas por Alfredo Margarido na controvérsia resultou a expulsão do casal de Angola (Seixas, 1989, p.138).

Não se pode deixar de reconhecer a posição excecional de Cruzeiro Seixas nesse período, não apenas por ser europeu, aparentado de um governador-geral, bem integrado na vida social, intelectual e artística local, reconhecido artista, ainda que controverso, mas realizando tarefas humildes, vivendo discretamente, sem constrangimento, a sua orientação sexual.

Seria inadequado, todavia, tentar encontrar analogias com o ambiente parisiense que James Clifford (1981) descreve em “On ethnographic surrealism”. A Universidade de Luanda ainda não tinha sido criada, a transferência das coleções para o novo edifício do Museu de Angola, inaugurado em 1956, dificultava a investigação e os trabalhos no terreno; a dinâmica intelectual em Luanda era incipiente e, para além da organização de exposições e do convívio com o casal Margarido, não houve articulação criativa de Cruzeiro Seixas com outros artistas e literatos, antropólogos e etnógrafos, mas sim a convergência cautelosa de intelectuais oposicionistas à situação colonial e ao regime político vigente. Na exposição de 1957, os críticos denegriram a exposição, tratando-a como uma excentricidade do “Dali dos musseques”; a dimensão surrealista tornava menos evidente a intenção de denúncia da situação colonial e permitia valorizar a cultura dos colonizados, colocando a par, nas salas da “instalação”, desenhos e esculturas de Cruzeiro Seixas e peças africanas colocadas em plintos.

Dois exemplos permitem perceber a complexa posição de Cruzeiro Seixas: em primeiro lugar, a amizade com o industrial Manuel Vinhas, figura cosmopolita, mecenas de artistas e literatos, que detinha um importante grupo industrial em Portugal, colónias africanas e Brasil. Cruzeiro Seixas passou a aconselhá-lo na aquisição de obras de artistas contemporâneos portugueses, e, dando-lhe a conhecer as peças etnográficas, entusiasmou-o, passando também a recolher-lhe peças. Há, no espólio de Cruzeiro Seixas na Biblioteca Nacional de Portugal, um relatório de uma viagem ao interior de Angola, na zona de Malange e Bié, destinada a pintar em árvores painéis publicitários da cerveja Cuca, produzida por uma das fábricas de Vinhas; no relatório, Cruzeiro Seixas descreve sucintamente os trajetos, apontando, além dos sítios em que colocou a publicidade, as ocasiões, os locais, e as despesas feitas para a aquisição de peças etnográficas (BNP ESP N38/854-855, 1960).

O segundo exemplo é o da relação com os artistas e intelectuais locais, a quem, ao mesmo tempo, atraía o talento, a civilidade e a cultura de Cruzeiro Seixas, e incomodava a sua expressão artística, ousada e perturbadora. Disso é sinal o seu encontro com Albano Neves e Sousa, pintor tradicional de paisagens e de figuras de personagens locais: pouco entusiasmado com um quadro oferecido pelo então jovem artista moçambicano Malangatana, de passagem por Luanda, o ofereceu a Cruzeiro Seixas (Pereira, 2020, p.271-272).

Em 1960, com dificuldades em encontrar novos trabalhos, Cruzeiro Seixas é contratado para o Museu de Angola, instituição sobretudo de história natural, mas com secções de arte portuguesa e etnográfica. Apesar de a sua função ser formalmente a de carpinteiro, Cruzeiro Seixas irá ter atribuições de curadoria e museologia, definindo uma nova disposição das salas e organizando várias exposições de pintura e etnografia (Seixas, 1989, p.137), de surpreendente qualidade museológica.

Uma das tarefas então realizadas foi a organização da sala para ser exposta a coleção de pintura portuguesa contemporânea que Manuel Vinhas, por sugestão de Cruzeiro Seixas, depositou no Museu de Angola. Em lugar de destaque estava o quadro de Malangatana. Por essa ousadia, é interrogado de novo pela polícia política.

Com o início das insurreições armadas de libertação, durante o ano de 1961, o ambiente geral em Angola torna-se belicista, e Cruzeiro Seixas, recusando-se a pegar em armas, sente a necessidade de regressar, ele e os pais, a Portugal. Não tendo outros recursos, propõe a Manuel Vinhas que lhe compre a coleção etnográfica, o que este acede, apesar do preço elevado que o artista lhe pede (BNP N38/101, s.d.).

Assim, a mostra de máscaras e os instrumentos musicais que Cruzeiro Seixas organiza no Museu de Angola em 1964, igualmente numa lógica museológica inovadora, incluía peças que eram suas mas que se tornaram, entretanto, de Manuel Vinhas. A notícia de *A Província de Angola* indica, na ocasião, a proveniência e o tipo das peças:

Os espécimes escolhidos são propriedade do Museu de Angola, do Museu do Dundo e do Museu da Huila, e dos srs. Manuel Vicente e António Frade. O público pode ali apreciar 19 cordofones, um membrancordofone (*jimba*), 35 membranofones, 107 idiofones, 58 máscaras e dois capacetes para dança, objetos esses recolhidos em diversas regiões da Província, principalmente em Luanda, Malange, Maquela, Dundo, Cuango, Ganguelas, Lunda, Chicapa, Luchimo, Bailundo, Duque de Bragança e Alto Zambeze (BNP N38/2231-A, s.d.).

No texto que redigiu para a exposição, Cruzeiro Seixas procura enfatizar o valor artístico das peças que eram expostas, máscaras e instrumentos musicais. Na sua análise, a subtil qualidade formal das peças não pode ser separada da *performance* dos instrumentos:

Se nas cubatas, (ou em geral em todos os objetos pintados através de toda a África) raramente são empregadas cores puras e antes subtis combinações, sem margem para acasos, a fragilidade de um *quissange* [um lamelofone] (e sendo o *quissange* tido por diversas autoridades como único instrumento talvez inteiramente inventado pelo africano, o que melhor corresponde às suas subtis *nuances* rítmicas, ao seu tipo de musicalidade), ou de um *cacoche* [violino de uma única corda] só é audível a poucos passos do tocador, recorrendo este, sabiamente, à cumplicidade da noite. Quando a música negra se ergue, possibilitada por outros seus instrumentos, mais vibrante, não seria ainda de bárbara que a classificaríamos, mas sim como coisa intensamente dramática, lembrando um teatro perdido. Esse destino teatral é, de resto, exibido hoje, dramaticamente, no grande palco internacional (BNP N38/2083, s.d.).

A consideração das peças e a da arte africana conduz necessariamente à questão da “civilização africana” na medida em que, apesar do seu irreconhecimento e secundarização, “cada vez mais o homem procurará nos mais herméticos vestígios de outras civilizações as raízes necessárias à sua estatura. Esses contactos equivalem ao poder evocador do sonho, acordado ou adormecido, proposto pela psicanálise e festejado pelos surrealistas – o distante guardião espiritual dos índios das planícies” (BNP N38/2083, s.d.).

A consideração de uma dimensão “mágica” das peças não é referida, mas é posta em correspondência com o sentido misterioso do existir a que poetas e pensadores europeus foram sensíveis. “Há muito de maldito nestas artes ditas selvagens, maldito como a poesia de Hugo, Novalis, Baudelaire, Lautréamont, Rimbaud, Malarmé: esse é o seu lado mágico, ou aquilo que lhe pôde ser atribuído pelo nosso desconhecimento europeu (esquecimento ou recalcamento) de tais formas e símbolos” (BNP N38/2083, s.d.).

E se atualmente a arte e o pensamento africano já não podem ser ignorados, eles são “mais uma grave advertência a lembrar que, só depois do homem se readmitir todo, poderá realmente criar uma moral, não só à medida dos seus medos, mas à medida das suas necessidades humanas, e das aquisições últimas da sua inteligência” (BNP N38/2083, s.d.).

Manuel Vinhas, depois de adquirir a coleção a Cruzeiro Seixas, lançou o projeto de criação de um Museu de Arte Tradicional em Luanda, juntamente com outro de arte contemporânea, este inspirado no Museu de Arte de São Paulo Assis Chateaubriand (São Paulo, Brasil), para o qual pediu a um ateliê de arquitetura que elaborasse um projeto para instalar a coleção etnográfica num edifício próprio. Quando os arquitetos lhe indicaram o preço do projeto, que achou excessivo, informou a Cruzeiro Seixas que iria procurar alternativa. Não se sabe se houve seguimento desse processo, tem-se tentado identificar o ateliê e localizar o projeto. Quanto à coleção adquirida, ela terá ficado depositada desde a sua compra, uma parte nas reservas do Museu de Angola, outra nas instalações da Associação Desportiva e Cultural da Cuca, sua proprietária nominal. Não havendo um inventário dessa coleção, apenas conhecemos dela duas dezenas de fotografias de peças individuais ou grupos de peças, guardadas no espólio de Cruzeiro Seixas (BNP ESP N38/1608-1626, s.d.) e imagens de partes do conjunto, que apareceram numa reportagem da revista *Notícia* (BNP N38/2130, 1964). Em carta de um amigo que o ajudou, refere-se à arrumação das peças, cerca de oitocentas a mil, num armazém (ESP CS-D.A./CORR.REC.01.50, s.d.).

O destino da coleção é obscuro. Durante a guerra civil, que se iniciou em 1975, ambas as partes da coleção foram saqueadas, e, de acordo com informações informais obtidas junto da família Vinhas, apenas encontraram peças soltas e danificadas nas caves do Museu de Angola. Após a independência de Angola, em 1975, o governo do Movimento Popular de Libertação de Angola, que controlava Luanda, privatizou a empresa Cuca e vendeu-a em 1981 a um grupo francês. Pedidos de informação junto do Museu de Angola, do Museu de Antropologia e da empresa Castel, proprietária atual da marca Cuca, sobre o paradeiro da coleção não obtiveram resposta.

Regressando a Portugal em 1964, Cruzeiro Seixas recebeu uma bolsa da Fundação Calouste Gulbenkian em 1967, o que lhe permitiu viagens pela Europa e colmatar o conhecimento dos principais museus europeus de arte e etnográficos. A continuidade do seu interesse pelo Museu de Angola e a expectativa de criação de um museu em Luanda dedicado à “arte tradicional” mostram-se no texto “Das térmitas e dos homens” (Seixas, 1967), publicado em 1967 no *Boletim Cultural da Câmara Municipal de Luanda*, em que alude às suas visitas recentes a museus etnográficos em Paris, Londres e Genebra.

A partir de 1967, começou a expor em importantes galerias em Lisboa e no Porto, depois fora do país, afirmando-se progressivamente como artista. Todavia, a situação que encontrou em Portugal após o seu regresso desiludiu-o; impressionava-o, mesmo entre artistas e intelectuais, a falta de atenção e sensibilidade às questões de África, fosse à brutalidade do colonialismo e à violência das guerras de libertação, que conduziam ao desaparecimento de uma “civilização”, fosse aos conflitos internos e às formas neocoloniais que sucederam às independências das antigas colónias. Também a sociedade europeia lhe parecia crescentemente sob o domínio do económico, promovendo uma vida vertiginosa e alienada de si.

Após ter concluído o período de bolseiro, esgotado o rendimento da venda da coleção africana, vivendo sempre no limite da sobrevivência, Cruzeiro Seixas começou, em 1970, com o apoio de Vinhas, a exercer funções de programador em importantes galerias de Lisboa, do Estoril e do Algarve, possibilitando a realização de exposições, com artistas jovens, internacionais, também africanos, que começavam a expor em Portugal, como o moçambicano Inácio Matsinhe. A memória de África manteve-se, e, em 1975, expôs na Galeria da Emenda guaches do período em que viveu em África. Disse na ocasião: “Esses guaches, feitos nas minhas rápidas viagens ao interior de África (e ao meu interior), eram como cartas de amor, trocadas entre mim e esse continente extraordinário. Cartas de um amor correspondido, poderia dizer” (BNP N38/923, 1975).

Prosseguindo também a criação poética, Cruzeiro Seixas continuou a assinar os seus poemas como tendo sido escritos em África, ainda que a data aposta fosse muito posterior à do seu regresso (Seixas, 2020).

Apesar de um período economicamente difícil nos anos 1970 e 1980, Cruzeiro Seixas continuou a expor regularmente em Portugal e fora do país, mostrando a sua vasta criação de pintura, desenho, colagem, escultura e cenografia. Cruzeiro Seixas prosseguiu também o cultivo da poesia, publicou diversas obras, que o tornaram figura incontornável da poesia surrealista em Portugal. Numa entrevista dada em 1985 a Bernardo Pinto de Almeida, académico e poeta, Cruzeiro Seixas assume-se antes de mais nada como um “poeta que desenha”, pois o poeta é aquele que enfrenta a singularidade misteriosa do mundo, o que ele pode apreender em “África”.

Como foram os seus anos de África?

Fiz de tudo, nesses anos fabulosos, 14 no total. Fui corretor de seguros, vendi rádios no mato, que eu adorava, de outra vez o Manuel Vinhas arranjou-me pintar nos imbondeiros a palavra ‘CUCA’ que era aquela marca de cerveja, enfim fiz de facto de tudo. Sabe? Eu sou um selvagem, e esse foi o grande período da minha vida (BNP N38/2394, 1985).

Mas, na história recente do mundo, “África” como possibilidade de experiência de mistério e solitude “selvagem” já não existe: “E hoje já não há África. O mundo subitamente tornou-se muito pequeno. … Em África havia o desconhecido, essa transbordante dose do que não se sabe, desconhecido, misterioso, profundo do transcendente. Esta civilização racionalista mete nojo!” (BNP N38/2394, 1985).

Se o surrealismo constituía uma ética, na medida em que propunha um aprofundamento do conhecimento de si, antes de uma intervenção na sociedade, na radicalização que prosseguiu, mesmo o surrealismo se supera e sublima: “Nunca me afirmei como surrealista. Sempre preferi que fossem os outros a dizer se o sou ou não. E alguns vão dizendo que sim. O que posso dizer do surrealismo é que foi a única filosofia com que me identifiquei e que para mim até hoje não foi substituída por qualquer outra tão moderna, tão atual” (BNP N38/2394, 1985).

Cruzeiro Seixas descobre-se em África, e África passa a ser o lugar matricial da sua experiência, de uma experiência direta de liberdade. Os seus poemas exprimem o duplo regime de “África”, como símbolo da equivocidade ontológica do mundo: por um lado, índice do constrangimento e da violência; por outro, experiência do misterioso e ocasião de liberdade.

Na extensa obra visual posterior a 1964, não há referências evidentes a África; apesar de se perceber, sobretudo nos desenhos dos anos 1960, a continuação da denúncia colonial e do autoritarismo político, não se identificam figuras que pudessem ser reconhecidas como aludindo a África. A experiência decisiva das “Áfricas” permanece nos decénios seguintes nos poemas; depois, até esse hábito esmoreceu, como se verifica em livros publicados posteriormente, permanecendo apenas nos seus “diários não diários”, um vasto conjunto de diários gráficos, criados desde os anos 1990 até ao seu falecimento, em geral pequenos dossiês reunindo por argolas folhas soltas, surgindo frequentemente na composição imaginativa de desenhos, textos, colagens de fotografias e diversos materiais (Cuadrado, Oliveira, 2021, p.183-263).

Se a venda da coleção constituía evocação penosa, último recurso para assegurar o regresso, seu e de seus pais, a Portugal, após o início da guerra em Angola, Cruzeiro Seixas guardou até ao fim algumas poucas peças de que não se quis separar (numa situação de aflição económica, depois de abril de 1974, foi obrigado a vender algumas peças, por meio de um antiquário). Depois, quando ganhou maior disponibilidade, voltou a reconstituir uma pequena coleção de peças etnográficas, não apenas exclusivamente africanas, adquiridas a comerciantes e antiquários, ou por permutas de obras suas que compuseram até ao seu falecimento a decoração das casas de sua residência.

## Eduardo Nery

De modo semelhante a Cruzeiro Seixas, os contactos com a cultura e a arte africanas foram, para Edurado Nery, um momento decisivo do seu percurso artístico, mas, em vez de Cruzeiro Seixas, a constituição de uma coleção de arte africana não marcou a sua juventude, ocorrendo tardiamente, no período da sua maturidade artística, gozando já de reconhecimento e estabilidade económica. Também diferente era a situação portuguesa, com os modos políticos e económicos europeus plenamente adotados, e já ultrapassados não apenas o Estado Novo e o colonialismo, mas também a descolonização, o regresso dos colonos portugueses e a instabilidade política posterior à Revolução de 1974.

Dezoito anos mais novo que Cruzeiro Seixas, Eduardo Nery formou-se nos anos 1950, ainda no Estado Novo, mas num contexto político em que os intelectuais oposicionistas não procuravam subverter os modos morais e intelectuais dominantes, como o surrealismo anos antes. Assumiam, em vez disso, preocupações de progresso social e económico em que seria possível recuperar e modernizar modos tradicionais. Formado em artes pela Faculdade de Belas-Artes de Lisboa e com frequência incompleta do curso de arquitetura, as suas primeiras obras cultivaram o gestualismo, influenciado por Hans Harnung e Pierre Soulages. Liberto do serviço militar, por razões de saúde, e, por isso, dispensado de combater nas colónias, Nery estudou tapeçaria em França com Lurçat, tomando conhecimento da amplitude e da diversidade dos caminhos da tapeçaria contemporânea.

Regressado a Portugal, desenvolve, a partir de 1965, obras com um estilo próximo da *op art*, trabalhando as variações cromáticas, a repetição de padrões e os jogos cinéticos. Interessado na desmontagem da percepção visual, concebe uma pintura que se expande tridimensionalmente, criando múltiplas dimensões, de extremo rigor geométrico. Criando universos perturbadores, à beira de uma ameaça ou atravessadas por uma intensa ironia, eles conjugam mistério e humor, suspense e ironia, numa desconstrução formal das imagens que joga com repetições e *trompe d’oeil*.

Não abandonando a paisagem abstrata inserida numa dimensão cósmica, explorou, a partir dos anos 1970, colagens, fotografias e muitas outras modalidades artísticas: o azulejo, o vitral, a tapeçaria, o *design*.

Após a Revolução de 1974, colaborou em vários projetos de habitação social e foi escolhido para intervir artisticamente em numerosas obras públicas – bairros sociais, escolas, praças, jardins, instalações fabris, estações de comboios, também em espaços privados: casas particulares, escritórios, igrejas.

Implícita na amplitude e riqueza de linhas artísticas e das interligações com outras áreas, cultivou um conhecimento apurado da história da arte e da ciência, também um interesse por conceções do mundo e sociedade diversas da europeia, adquirindo, desde o final dos anos 1950, peças de culturas extraeuropeias, sobretudo em feiras de segunda mão, e foi reunindo livros especializados. Como explicou em várias entrevistas, tinha um interesse difuso pela religiosidade humana, reconhecia um valor intrínseco em peças de arte e na música, cuja proximidade apreciava no momento da sua própria criação (Fernandes Dias, Nery, Bastos, 2009, p.62). Esse interesse não era apenas focado em África, mas em peças cuja qualidade estética era reconhecida pela sua acuidade artística e lhe despertavam interesse intelectual. Assim, quando nos anos 1990 foi convidado a criar um extenso painel de azulejos no Aeroporto de Macau, então em construção, teve a possibilidade de ali aprofundar o seu interesse pela arte chinesa.

As razões da sua preferência pela arte africana foram de algum modo circunstanciais, resultante de uma crescente oferta de peças em feiras, em lojas de antiquários e por comerciantes, a partir de meados de 1990, num período de desafogo económico cujo momento culminante foi a realização da Exposição Universal de Lisboa, em 1998.

Tal como a de Cruzeiro Seixas, a formação da coleção dependeu apenas dele próprio, mas de modo mais informado: se inicialmente foi escolhendo peças do seu agrado, por vezes de menor qualidade, depois a seleção foi-se apurando, alargando-se a peças de melhor qualidade e mais raras. Em poucos anos, a coleção de Nery estendeu-se a mais de mil peças, de 153 etnias dos povos subsarianos – esculturas, cerâmicas, tecidos, peças de mobiliário e instrumentos de música –, e destacando-se as máscaras e esculturas de Camarões, Guiné-Bissau, República Democrática do Congo, Costa do Marfim, Nigéria e Guiné. Cada uma das peças era minuciosamente analisada e descrita, tanto nas suas características materiais como na sua função e no seu simbolismo, criando-se entre o artista e as peças da sua coleção uma proximidade que era necessária à sua própria criação.

Se o seu sucesso artístico o tornou alvo de curiosidade pública, referenciado em revistas de decoração e acontecimentos mundanos como um “colecionador de arte”, as imagens da sua residência familiar, um apartamento burguês no centro de Lisboa, mostravam quadros seus combinados com obras e mobiliário europeu – estatuária medieval, mobiliário do Renascimento, entre outros –, também, depois da sua estada em Macau, com peças chinesas, sobretudo mobiliário e cerâmica. A coleção africana não estava ali presente, mas acumulava-se em todos os lugares possíveis do seu ateliê, num outro lugar da cidade, junto da sua rica discoteca de música clássica, *jazz* e de música etnográfica. Ali, em entrevistas para revistas de arte e reportagens televisivas, Nery mostrava-se rodeado da sua coleção – estatuetas, máscaras, tecidos etc. que se agrupavam no chão e em prateleiras, misturados com materiais de pintura e quadros acabados.

Eduardo Nery foi apresentando a sua coleção africana em várias exposições, em alguns casos a par de obras suas, outras só de peças etnográficas, elaborando para algumas delas catálogos com análises pormenorizadas das peças apresentadas: em Esposende, “O eterno feminino: a mulher na arte africana” (Câmara Municipal..., 2006), na Câmara Municipal de Esposende; no Museu d’Arte de Fão (2006); no Maihaugen Norges Olympiske Museum, em Lillehammer, Noruega (2007); no Musée Théodore Monod d’Art Africain, Dakar, Senegal (2007); sobretudo, na grande exposição “Formas e energias”, na Câmara Municipal de Lisboa, no Páteo da Galé (2009); e, três meses após o seu falecimento, a exposição “Função, estética e ciência”, no Instituto de Investigação Científica Tropical (2013).

Do estudo, da análise crítica e da interpretação das peças resultou a publicação de *As artes africanas: aproximação estética de um artista*, em 2010, em que a caracterização das correntes estéticas lhe permitiu apontar os modos como, por meio dos gestos, posturas e materiais, o homem, o cosmos e a religiosidade se intersectam na diversidade das artes africanas.

Nessa obra, e nos catálogos das apresentações da sua coleção, Nery dedicava a cada uma das peças uma análise minuciosa, conjugando observações antropológicas e estéticas. Tomemos como exemplo o comentário sobre uma estatueta de um *Fon*, da etnia Bangwa, recolhida nos Camarões:

A estrutura figurativa deste *Fon* (ou juiz?) em reflexão é muito elementar e muito pouco realista, tirando partido sobretudo das deformações anatómicas, com um braço maior do que outro, um longuíssimo pescoço e as pernas muito atarracadas e sem pés, que se confundem com o banco. Aliás, trata-se de uma obra em que cada parte do corpo foi tratada separadamente, ‘como tenho encontrado em muitas obras de arte africana’. A cabeça e a cara com olhos salientes são um pouco toscas, mas muito expressivas, realçadas pela textura encrostada, que se estende a todo o corpo, numa grande variação de tons de castanho. Também é de realçar o jogo rítmico dos muitos anéis que essa figura ostenta, que se contrapõem à serenidade e à reflexão dessa pose. A cabeça é interessante vista de perfil e de frente, mas é muito simplificada vista de trás. Esta postura, “evocando longinquamente ‘O Pensador’ de Rodin”, simboliza prudência, inteligência e sabedoria, qualidades que se esperam do rei ou do chefe (Fernandes Dias, Nery, Bastos, 2009, p.153; destaques acrescentados).

A análise, feita na primeira pessoa e estabelecendo paralelismo com a arte ocidental, combina a minúcia com o sentido geral de composição. Análogos movimentos se encontram na sua própria criação artística. Eduardo Nery ora segue um caminho de afastamento, pesquisando os modos extremos do “teatro do olhar”, seja “o da construção puramente ótica da imagem, seja o da sua transfiguração semântica”, como sugere Fernando de Azevedo na apresentação da retrospetiva da sua obra (Azevedo, 1997, p.24), ora sobrepõe as peças africanas às europeias, procurando a expressão de uma interioridade anímica e de um sentido cósmico, mesmo que grotesco.

Coetâneos da sua recriação de “máscaras” – guaches de cores intensas que denomina de “pintura expressionista”, apresentados na exposição “O outro lado do rosto/o visível e o invisível”, na Galeria Valbom, em 2003 –, Nery expôs séries de fotomontagens com máscaras africanas. Na primeira, “Transfiguração”, de 2003, as máscaras africanas surgiam cobertas de véus transparentes. Como o historiador de arte José-Augusto França (2006, p.20) aponta, no catálogo da exposição, “[pela] realidade geométrica do seu pensar sensível, ele é levado a opor uma irrealidade de espírito, em que outro pensar se assume. Homem e cosmos andam separados no Ocidente, por metáfora nossa, sem remédio; para compreendermos a necessária totalidade temos que nos imaginar fantasmas”.

Em “Metamorfoses”, exposição de 2005, na Fundação Calouste Gulbenkian, as fotomontagens de máscaras africanas fundiam-se com rostos europeus de homens e mulheres.

Eduardo Nery mergulha no inconsciente, na ausência de tempo que é o recomeço eterno da rede múltipla de aspetos que formam o ser humano, captado no diálogo entre o belo e o monstruoso, entre o real e o aparente. O artista cria, deste modo, um objeto de linguagem que se afirma, no entendimento de Adorno, como um ‘estremecimento de terror do mundo primitivo’, como escreve Ana Maria Gastão em *Poéticas da Ironia* (Molder, Gastão, 2005, p.114).

Para Eduardo Nery, África não foi, como para Cruzeiro Seixas, uma realidade diretamente vivida, mas antes uma entidade suposta como o horizonte desde o qual surgem as peças que adquire, coleciona, estuda e expõe. Surge como uma entidade concebida a partir das obras de história de arte e de antropologia que consulta, por meio da vivência imaginada dos criadores e dos utilizadores das peças, também, talvez, dos comerciantes a quem adquiriu as peças. É uma África sem história, sem a duplicidade intrínseca e a dramaticidade que se encontra em Cruzeiro Seixas; os problemas que coloca são circunstanciais – as dificuldades sentidas pelos comerciantes devido a perturbações internas nos seus países (Dias, 2009, p.31) – ou aqueles que, no quadro das disciplinas a que recorreu, lhe convinham: as intrínsecas qualidades estéticas e simbólicas de cada peça, a distinção entre o original e a cópia, a questão da autenticidade das obras que não foram utilizadas em rituais, mas produzidas para venda (p.55).

No vasto campo de pesquisas de Eduardo Nery no modernismo artístico – da fotografia à arte pública, da sua sensibilidade, da geometria ao expressionismo, dos seus interesses artísticos, da música clássica à arte africana – surgem, contrapolares, a “abstração” e a intensificação da experiência “sensorial”, de que resultam desconstruções, ironias, sobreposições e anamorfoses.

Houve, todavia, algo de problemático nessa relação com a arte africana. No longo diálogo com o antropólogo José António Teixeira Dias, transcrito em *Formas e energias,* catálogo da exposição da coleção no Pátio da Galé, em Lisboa, em 2009, o fascínio pelas obras africanas teve um impacto não apenas no artista e na sua criação, mas na vida familiar; as alusões são discretas, mas suficientemente claras e permitem perceber a resistência da família à compra das peças, que passaram a ter um peso económico incomportável (Dias, 2009, p.48). Num texto que a viúva, Maria da Graça Nery (2013, p.10), também artista plástica, redigiu para o catálogo da exposição póstuma, percebe-se a intensidade da relação do artista com a coleção e a preocupação nos últimos anos pela sua preservação: “Foi uma paixão arrebatadora durante tantos anos, uma paixão cada vez mais intensa nestes últimos! A tua coleção era como se fizesse parte de ti e tu dela!”.

Após o seu falecimento, todavia, a família viu-se obrigada a vender a coleção, conjuntamente com a biblioteca especializada, ao empresário e colecionador José Berardo, que a integrou na sua já vasta coleção de arte (Berardo…, 14 jul. 2017).

Qual a “África” que Eduardo Nery persegue por meio da sua coleção? Inserida historicamente num momento posterior à descolonização, a visão que a constituiu pode ser considerada “pós-colonial”: não há no intento da coleção e na escolha das peças qualquer ressonância da colonização portuguesa; em vez de as diminuir ou depreciar, a sua atitude é de exaltação e deslumbramento, não hesitando, como vimos, em comparar peças africanas com exemplos maiores da arte ocidental. Não se encontra em Eduardo Nery nem uma visão “colonial”, que distinguisse os registos de avaliação das peças africanas analisadas das da tradição da arte ocidental ou das suas, também não uma visão, que veremos em José de Guimarães, que considerasse a “debilidade” das peças ocidentais em comparação com a “forte carga mágica” das africanas, nem ainda, por fim, os prenúncios de uma visão “decolonial”, que descontruísse os supostos estéticos da sua mesma avaliação estética e integrasse a modernidade do seu olhar na articulação de modernidade, colonialidade e decolonialidade apontada por Mignolo e Walsh (2018, p.135-152).

Assim, as referências das suas “máscaras” às máscaras africanas, ou, nas fotografias, a utilização anamórfica das peças da sua coleção, despoletam o sentido, não de um inteiramente “outro”, mas a possibilidade de recuperar, por esse “outro” e na criação artística, uma identidade antropológica mais funda, latente ou mesmo perdida, mas não alheia à consciência europeia – numa disposição diversa da radicalidade antropológica e ética de Cruzeiro Seixas. No encontro com as peças africanas, que quis compulsivamente renovar, Eduardo Nery procurou explorar dimensões que no Ocidente estão cindidas – o visível e o invisível, o consciente e o inconsciente, os vivos e os mortos –, alcançar um sentido de totalidade, um “mesmo” englobante, que as suas últimas criações em vida, pinturas de intensos círculos cromáticos, apresentadas na exposição “Sol e outras pinturas (2012-2013)”, na Fundação EDP, em 2014, parecem querer ilustrar.

## José de Guimarães

A situação de José de Guimarães é ainda diversa da de Cruzeiro Seixas e de Nery. Seu interesse pelas peças africanas surgiu num momento em que, oficial do Exército português, se encontrava em Angola numa comissão militar.

Nascido em 1939, José de Guimarães, nome artístico que tomou José Maria Fernando Marques da cidade natal, ingressou na Academia Militar e no curso de Engenharia na Universidade Técnica de Lisboa em 1957, iniciando ao mesmo tempo uma formação artística, com Teresa Sousa e Gil Teixeira Lopes, e estudando gravura na Sociedade Cooperativa de Gravadores Portugueses. Entre 1961 e 1966, viajou pela Europa para conhecer as obras dos mestres de referência nas tendências recentes, como a *pop art*, de que se aproxima. Oficial de carreira, engenheiro de transmissões, fez duas comissões militares em Angola (1967-1970 e 1970-1974), após o início das sublevações nacionalistas em 1961.

Conseguindo conciliar o serviço militar com a criação artística, muito influenciado pela *pop art*, apresentou-se em várias mostras e realizou diversas exposições, em Luanda, de gravuras e esculturas, recorrendo a caixotes e cabines telefónicas.

O contacto no terreno com as populações teve nele um impacto profundo, o reconhecimento de uma alteridade misteriosa: “Ao confrontar-me com aquele novo continente, o choque cultural não poderia ter sido maior. Porém, depois de uma desadaptação inicial surgiu um profundo interesse pelo conhecimento dessa nova cultura, embora distante da minha, ocidental, quer nas formas de atuação, quer nos seus conceitos” (Guimarães, 2009, p.89).

Numa disposição muito diversa do “amor correspondido” de Cruzeiro Seixas e do fascínio antropológico e obsessão analítica de Eduardo Nery, “África” surge para José de Guimarães como um problema de “comunicação”. Reconhecendo-lhe decerto o fundo misterioso, o que o impressionou foram as suas potencialidades comunicativas:

Ao contrário da arte ocidental, o forte conteúdo mágico-religioso da arte tribal transforma os objetos artísticos em obras de arte utilitárias que se destinam à prática de rituais. Em geral, são esculturas de figuração muito explícita, umas vezes mais misteriosas que outras, em virtude de determinados ingredientes – espelhos, pregos, ossos – passavam a conter uma forte carga mágica ou, como as tatuagens, por exemplo – bem características dos povos africanos, inscrições no corpo humano com intenções nitidamente mágicas, ou ainda as pinturas de sinais que, na pele dos homens, têm o poder de propiciar vitórias na guerra, e nas mulheres podem ter a função de invocar fertilidade etc. (Guimarães, 2009, p.89).

Nesse contacto inicial, a cultura africana manteve-se, todavia, fechada, sentindo-se Guimarães incapaz de entender as peças com que se deparava, a vivência e os valores daqueles com quem contactava. Tal desconforto levou-o a uma nova fase, com a leitura de estudos etnográficos e antropológicos,

com a ajuda de amigos etnólogos, pude debruçar-me sobre o estudo da etnografia africana, da angolana e da arte tribal em geral, o que me permitiu chegar a um certo conhecimento do conteúdo das manifestações artísticas africanas que se apoiam em arquétipos culturais e sociológicos diferentes, ou até opostos aos meus. Recordo apenas alguns nomes que me ajudaram nessa pesquisa: Mesquitela Lima, José Redinha e padre Carlos Estermann, da Huíla, com quem tive o privilégio de conversar algumas vezes (Guimarães, 2009, p.91).

Decisivo foi o conhecimento das tampas das panelas Ngoyo, que

[se] transformam em verdadeiros processos de comunicação – comunicação ideográfica, que funciona como se de verdadeira escrita se tratasse. Os Ngoyos comunicavam[-se] entre si através de sinais ideográficos, inscritos em tampas de utensílios domésticos – terrinas –, uma espécie de altos-relevos com significados diversos, conforme a imagem utilizada nas suas mensagens, como se fossem provérbios de forte conteúdo moral, aliás muito mais eficazes do que porventura seriam se utilizassem a expressão oral (Guimarães, 2009, p.91).

Regressado a Portugal em 1970, nesse mesmo ano foi mobilizado para uma segunda comissão em Angola, que demorou até 1974. Então, já na posse de um certo conhecimento sobre arte tribal, propôs-se conceber um modo de comunicação de entendimento, um “alfabeto africano”. Não houve exatamente uma apropriação, mas aprendizagem do processo:

A arte negra fez-me saber como se efetua a concentração do significar e da carga mítica das formas. E, assim, na minha pintura, a forma passou a ser símbolo e um agente de grande poder atuante. Vi, em África, como eram usados os símbolos e para que serviam. … Apropriei-me de uma parte da sua utilidade: mais do seu ritual do que dos próprios significados (Guimarães, 2009, p.91).

Esse novo alfabeto, composto de 140 sinais, inteiramente convencional, teve uma génese demorada:

A sua génese demorou dois anos (1970-1972). Anos de angústia e de intensa convivência com o mundo africano. … As formas que lhes dei, essas, foram nascendo geradas e multiplicadas segundo o ritmo das circunstâncias e a necessidade de ampliar um vocabulário. Com símbolos, uns substantivos, outros adjetivos, uns emancipados, outros dependentes. Uns a gerarem os outros (Guimarães, 2009, p.91).

O conjunto de signos, ideogramas, que incluem rostos humanos esquemáticos, setas, linhas onduladas, gestos, sequências de números etc. constroem um grafismo que possibilita a sua recomposição e combinatória.

Descrevendo a sua conceção como que uma iniciação, dolorosa, mas necessária e profícua, foi esse o momento decisivo do seu percurso artístico. Só depois dele houve possibilidade de diálogo e, com ele, a possibilidade de compreensão com “esse mundo africano de cultura poderosamente intuitiva e que se exprime com formas carregadas, todas, de um grande poder de intervenção” (Guimarães, 2009, p.89). O alfabeto passou a ser um modo de compor “uma arte que já não era a arte de um pintor europeu nem de um pintor africano, mas uma arte miscigenada de duas culturas” (p.93), matriz do seu percurso posterior.

Regressado a Portugal, começou, após frequentar cursos de formação em Inglaterra, a expor com enorme sucesso em Portugal e na Bélgica, ganhando rapidamente repercussão internacional. A iniciação por que passou e a criação das primeiras obras mestiças permitiram-lhe aplicar, nos anos seguintes, método semelhante a outras linguagens pictóricas: a artistas europeus – Rubens, Velasquez, Leonardo; a figuras e elementos da história e da cultura portuguesas – às chamadas “Descobertas”, a Luís de Camões e a Fernando Pessoa; aos desportos; às paisagens portuguesas; à escravatura; às culturas mexicana, chinesa e japonesa, num processo aberto a múltiplas repetições, reformulações e combinações.

Cada uma dessas culturas estimulou-o a desenvolver uma linguagem comunicativa, um universo imaginário que procura conjugar a história e a arte portuguesa e europeia com as de países longínquos, numa tentativa de criação de uma “arte global”, em que o alfabeto africano se tornou “uma espécie de matriz ou trama, na qual se desenvolvem e progridem outros símbolos e códigos” (Guimarães, 2009, p.91).

O colecionismo de José de Guimarães acompanhou a sua maturação artística. Entre 1967 e 1970, ele foi adquirindo algumas peças, que se poderia dizer de artesanato, criadas por artífices angolanos inseridos no Instituto de Trabalho de Angola, coleção de cerca de setenta objetos que, regressado a Portugal, doou a um museu da cidade natal: “Recordo que, aquando da minha primeira estada em Angola, fiz uma pequena coleção de arte tribal, hoje no Museu de Arqueologia da Sociedade de Martins Sarmento, de Guimarães” (Guimarães, 2009, p.93; ver também Registo…, 1972).

Depois, regressado a Angola, e, a seguir, já na Europa, continuou a colecionar:

Mas foi a partir dos anos 1970 que de novo comecei, lenta mas sistematicamente, a colecionar arte tribal não só de Angola, mas principalmente de toda a região centro-africana, nomeadamente da Nigéria, Camarões, Gabão, Mali, Burkina Faso, Sudão, Congo, Guiné, Gana, Costa do Marfim, Togo, Benim, e que continua até aos dias de hoje. Entre eles, Mambilas, Mendes, Kanacas, Tchokwes, Baulés, Yaurés, Cabindas, Farngues, Bagas, Punos, lgbos, Markas, Bambaras, Dogons, Kotas, Noks, lorubas, lakas, Dans, Jukuns etc., que hoje dialogam juntos nos meus ateliês lisboeta e parisiense, como se de templos se tratasse (Guimarães, 2009, p.93).

E, na medida das suas viagens e exposições em vários pontos do mundo, a “paixão por diferentes culturas” levou-o a alargar o seu colecionismo, acrescentando ao seu interesse pelas peças africanas, adquiridas em Lisboa, Paris ou Bruxelas, “objetos artísticos, seja da cultura pré-hispânica, da cultura chinesa arcaica” (Guimarães, 2009, p.93).

As razões do colecionismo são várias: de aprendizagem artística, de reconhecimento das outras culturas e de repetição, como artista, e, nas suas palavras, do “trilho dos navegadores de outrora”:

A minha intenção mais profunda enquanto colecionador não se reduz a uma mera atitude de colecionismo. Existe, para além disso, um lado de reconhecimento e respeito por outras culturas, enquanto português. Portugal sulcou os oceanos, encontrou novos mundos, misturou-se e criou mestiçagem – a minha obra artística tem, de certo modo, seguido o trilho dos navegadores de outrora, buscando e alimentando-se nas culturas de outras regiões da qual os portugueses de seiscentos foram precursores. É esse encontro e este respeito que me faz admirá-los e desejar vê-los de perto, através das suas manifestações artísticas e poder contemplá-las! (Guimarães, 2009, p.93).

De um modo que pode surpreender, José de Guimarães parece ter procurado, após o regresso à Europa e as independências das antigas colónias, assumir como sua a gesta marítima de Seiscentos, para a valorizar como encontro de culturas e possibilidade de mestiçagens, mesmo quando explicitamente alude à violência do colonialismo, como nas séries sobre a escravatura.

Decerto que, na sua disposição, é de África que vem o ensinamento artístico, atenção que depois alargará a outros lugares como Brasil, México e China. O impacto da arte africana não se evidencia apenas na criação dos “alfabetos”, de um conjunto primevo de símbolos que se combinam, repetem, jogam em cada pintura, ou, a rigor, em peças que são duplamente esculturas e pinturas, mas também em “relicários”, caixas que conjugam elementos vários, trazendo memórias e evocações. Esses “morfemas” possibilitam a conjugação de referências mítico-simbólicas, de memórias trans-históricas, de alusões à contemporaneidade – no uso de objetos contemporâneos e iluminação LED –, num intento de miscigenação global, histórica e cultural.

Nas peças, sobressai um sentido de transfiguração – por exemplo, pela sobreposição de figuras, que se metamorfoseiam e conjugam visibilidades e invisibilidades. Na aproximação do antropólogo Ramon Sarró, José de Guimarães, que se transfigura de engenheiro em artista, de artista em *bricoleur*, é um *trickster*, “transforma motivos africanos em arte ocidental, e vice-versa, africanizando a sua arte originalmente “ocidental”, atingindo uma osmose cultural absolutamente necessária, sem a qual nós, humanos, viveríamos em universos desconexos que não podem ser medidos uns contra os outros” (Sarró, 2018, p.166).

Mediador, oficiante de rituais, Guimarães é, para outro dos seus intérpretes, o curador Nuno Faria, o que “esconjura”, num processo mágico de tradução e contacto, que passa da obra para o próprio espetador. Leia-se a análise no catálogo de “Esconjurações”, apresentação de peças da coleção de um importante grupo bancário:

Por muito que nele se identifique um conjunto de formas arquetípicas, essas formas estão sempre em processo, ou fazem parte de um processo de tradução, de movimento constante, consubstanciando um pensamento que nunca é estático e que não se reifica em formas ou figuras – nesse sentido, há uma ideia transversal a todo o trabalho prosseguido pelo autor, que é a ideia de contacto. Contacto, que significa aqui a busca daquilo que é ‘outro’, que é materialmente, simbolicamente, diverso, intraduzível e infigurável (Faria, 2016, p.9).

Por meio de suas obras, ele pratica formas sublimadas de “canibalismo”, ligando crenças arcaicas e futuristas, num processo de “apropriação” e de “navegação”, mas também de redenção. José de Guimarães, oficiante e *trickster*, capaz de envolver culturas diversas e os próprios espetadores, conjugaria as tensões dramáticas do mundo, na procura de uma harmonia do passado e do futuro, do visível e do invisível. Artista assumidamente global, ele retomaria e renovaria o sentido secreto da história de Portugal, de um projeto esotérico e transcultural de “esperança”:

É um horizonte de esperança, de aproximação e de incorporação de um projeto místico transcultural que tem vindo a ser prosseguido por uma extensa e intensa linhagem esotérica ligada a esta terra fronteiriça e zona de irradiação a que chamamos Portugal – a do Padre António Vieira, Fernando Pessoa ou Agostinho da Silva (Faria, 2016, p.10).

Que experiência pois d’“África”? Ela corresponde a uma iniciação num modo de comunicação que coloca em “contacto” épocas e culturas que se ignoravam. Se interpretamos corretamente, a extensão das coleções a novas realidades geográfico-culturais, incluindo peças pré-colombianas e chinesas arcaicas, surgiria não apenas do “gosto” do artista, mas de uma intuição mais profunda. Essa intuição visava contactar a “ancestralidade” marcante dessas coleções com as histórias moderna e contemporânea, perpassadas por experiências de violência e desenraizamento na Europa, em África, nas Américas e na Ásia. Pretenderão os “alfabetos” evocar essa ancestralidade e traduzir o sofrimento que aliena a vida? Integrada em novos “relicários”, a “força”, que se transpõe da “ancestralidade” para um futuro “esperançoso”, transferir-se-ia pelo criador para a obra, eventualmente para o espetador, participantes de uma idêntica experiência de esconjuro. Se vemos bem, numa arte global concebida como exercício comunicacional, seria toda a experiência artística que se “africanizaria”?

## Considerações finais

A concluir, um breve *excursus*: na articulação das três coleções, um novo tópico emerge da pesquisa, a história dos projetos de museus de arte africana e as razões do seu fracasso. Em primeiro lugar, o Museu de Arte Tradicional, em Luanda, que seria criado por iniciativa de Manuel Vinhas, em edifício a ser criado de raiz, tendo como núcleo a coleção de Cruzeiro Seixas; quais as razões que impediram a sua concretização? Até que ponto se desenvolveu o projeto? O que aconteceu à coleção que deveria ser ali exposta após a independência de Angola?

Outro projeto malogrado que importaria melhor conhecer foi o de um museu de arte africana em Lisboa, anunciado em 2008, em que se empenharam altas figuras do Estado, visando reforçar a posição de Portugal nas relações entre Europa e África.

Apresentado publicamente como África.cont, o novo museu seria instalado num edifício que o arquiteto David Adjaye foi convidado a projetar, junto ao rio Tejo, em Lisboa. Como momento preparatório, realizaram-se exposições dedicadas às coleções etnográficas de José de Guimarães, José Berardo e Eduardo Nery, organizadas num lugar nobre de Lisboa, o Pátio da Galé, ao longo de 2009, pela Câmara Municipal de Lisboa (Coelho, 10 dez. 2008, 6 jan. 2009, 26 jun. 2009). Todavia, o projeto, que envolveria parceiros privados e entidades públicas, não chegou a desenvolver-se, em virtude da crise económica nacional e internacional, e foi abandonado em 2012 (Africa.cont., 28 jun. 2012).

Na sequência dessa suspensão, as coleções envolvidas seguiram diferentes caminhos: o empresário José Bernardo adquiriu, em 2017, a coleção de arte e a biblioteca especializada de Eduardo Nery e prosseguiu a instalação de partes da sua coleção de arte africana em espaços museológicos anexos a empresas suas: assim, no Hotel Monte Palace, no Funchal, no Palácio da Bacalhoa, em Azeitão, no Undergound Museum, em Anadia, e num projeto de museu em Estremoz, no Alentejo, que deveria igualmente acomodar a coleção de azulejos. José de Guimarães, em 2010, depositou uma parte significativa da sua coleção no Centro Internacional de Artes José de Guimarães, construído na sua cidade natal por ocasião da Capital Europeia da Cultura de 2012, opção que renovou em 2020 com novo depósito.

Histórica, cultural e intelectualmente, as três coleções dispõem-se num quadro restritamente ocidental, português e europeu, de considerar a “arte”, “África” e o colecionismo privado. Se insistimos sobre as especificidades de cada coleção, a complexidade das motivações dos artistas como colecionadores e a particularidade das suas relações intencionais com “África”, é porque a densidade de registos, aspetos e variáveis se perde ou se ilude por uma precipitação empírica ou preconceito conceptual. Há decerto no estudo de outras coleções que, para relativizar figuras, situações e processos, será necessário ampliar personagens, geografias e conceitos. Todavia, é por enfatizar a existência de vários liminares – nas motivações dos colecionadores, nos processos de colecionismo e nas próprias coleções –, por atender a detalhes e não descurar das equivocidades do que se prejulga óbvio, que o estudo se poderá mostrar profícuo na investigação futura.

## Data Availability

Não estão em repositório.
